# Annotations

**Published:** 1898-09-03

**Authors:** 


### Annotations.

### The Water Supply in East London.

It is much to be regretted that the *Times*, speaking through its "Special Correspondent," should make light of the failure of the water supply in the East of London. It may be true—we have no doubt it is—that even under the intermittent system of supply a very large amount of water is being distributed, but that does not take away from the gravity of the facts that the sanitary machinery of the district in question is organised on the basis of a constant service, and that a sudden reversion to an intermittent supply in a district unprovided with storage cisterns is attended with great danger from a public health point of view. To say, as the "Special Commissioner" does, that "when signs of danger appear it will be quite time enough to point to them"; and to add that "at present there are none, and no reason whatever to expect any," is equivalent to saying that the whole system which has been introduced at such enormous expense for the immediate removal of excreta by water carriage is useless, and that it does not matter whether the apparatus by which it is effected works well or ill. Every week landlords are being compelled to incur expense because the closets in their houses have an inefficient flush, and this on the ground that such closets are dangerous to health; it is, indeed, accepted as an integral part of the "water carriage" system that the apparatus must be kept constantly in good order; yet when by the failure of the water supply thousands of closets are reduced to the condition of having, for many hours of the day, no flush at all, we are told that there is no reason whatever to expect any danger! We are not here concerned to blame the East London Water Company, or, indeed, in view of the unprecedented drought, seriously to blame anyone, but we are concerned to protest against false teaching. It is quite possible that as improvements are effected in the construction of cisterns it may become necessary to review, and perhaps alter, some current notions as to the evils connected with their use; but so long as an enormous district remains without cisterns, and depends, for the cleanliness of its sanitary arrangements, upon the maintenance of a constant service, to say that there is no danger in cutting off the water supply for eighteen hours out of the twenty-four is absurd.

### Travel and Technical Education.

For one person who went abroad for a holiday even twenty years ago hundreds go now. Do we, then, know more of foreign countries than we did? So far as their scenery goes, perhaps we do; but with regard to people, customs, and habits of thought, it may be that we learn less than we did when we sat at home at ease, and did our travelling by the aid of the books written by visitors who spent a long time in each country they wrote about and made a study of its national peculiarities. A personally-conducted Cook's tour, supplemented by a study of Baedeker, gives one an external knowledge of a place; but it helps but little for anything deeper. It is almost impossible to learn much in any country without knowing the language. Would it not be worth while, then, for people to make up their minds in the autumn where next year's holiday is to be spent, and devote some of the winter's leisure to rubbing up the rusty French or German that will then be required? Then trips abroad need no longer be confined to one beaten track, where everybody speaks English, and you are told nothing that is not to found in the guide-book; but each one could go to some place which held a special interest for him. It would be possible to see something of foreign ways of living and working, and we could then judge if these were superior to our own, or adaptable to our circumstances. We should gain information that might very well be profitable to us at home, and the annual "wander-monat" would be more interesting as well as more profitable than is the ordinary round of castles, cathedrals, and hotels.

### Suburban Villas and Gravel Soil.

When a suburban jerry builder has speculated in a plot of land nothing delights him more than to find a little gravel thereupon, partly because it fetches a good price, but especially because gravel is commonly looked upon as a healthy soil. "Gravel soil" has, indeed, become such a fetish in the minds of many that it is well to draw attention to certain remarks upon soil in relation to disease made by Sir Charles Cameron at the Congress of Public Health lately held in Dublin. What he insisted upon was the fact that typhoid fever in Dublin showed an excessive incidence upon those portions of the city which stand upon gravel as compared with those which have a stiff clay for their foundation. It had, he said, long been assumed that the soil of Dublin lying nearest to the river was water-logged, and that this was the cause of the prevalence of fever in those parts. A few years ago, however, he had shown that the sub-soil water came much nearer the surface in the higher districts than in those near the river. The explanation was that the low-lying portion of the city stood on gravel, from which the water rapidly drained away, while the higher portions were on stiff clays, by which the water was retained. The prevalence of fever in the lower part of the town had, in fact, nothing to do with water-logging; what it had to do with was the fact that gravel is a porous soil which easily becomes foul, and that the typhoid bacillus requires air for its development, air which it can easily get in a damp gravel and not so easily in clay. A foul gravel is, indeed, a most dangerous soil on which to build. For warmth, for dryness, for absence of fog, and for facility of walking directly after rain, just when the air is at its purest and its best, there is nothing like gravel; but when gravel has been rendered foul by infiltration with organic matter, it may easily become a very hot-bed of disease, and in relation to this one must remember that those who dwell in suburban villas, and in houses built in rows, are apt to suffer from the sins of their neighbours so far as the subsoil is concerned. Where houses are planted at some distance apart, each standing in its own plot of ground, gravel is admirable; but whenever houses are so closely placed that the soakage from one may interfere with the ground air of another, gravel is but a doubtful blessing, its dangers being, of course, all the greater where the water supply is derived from wells.

